# Utilizing a Theory of Change for Better Health Outcomes

**DOI:** 10.3389/fvets.2022.929365

**Published:** 2022-06-30

**Authors:** Nadine Lamberski

**Affiliations:** San Diego Zoo Wildlife Alliance, Escondido, CA, United States

**Keywords:** health outcome, lagging indicator, leading indicator, outcome measure, quality of life, theory of change, veterinary medicine, zoological medicine

## Introduction

What we measure matters. The primary purpose of health care is to provide good patient outcomes. Health outcomes are defined as those events occurring as a result of an intervention and are measures of quality of care. A health outcome refers to both physical and psychological well-being and takes into account the length of life as well as the quality of life. Measuring outcomes helps make decisions about how to best care for patients. Measuring, reporting, and comparing outcomes are important steps in achieving better health outcomes. Improving health outcomes can improve the performance and accountability of health care teams by uniting the interests and activities of stakeholders around a common goal (1, 2).

The use of health outcomes and outcome measurements are imbedded within the human health care system. A key word search using the free search engine Pubmed confirmed the relative frequent use of health outcomes in the health care literature, the paucity of the use of the term in the veterinary literature, and the near lack of the use of outcome terminology in the zoological medicine literature (Table 1).

**Table 1:** The number of total citations returned following a key word search using the search engine Pubmed on 15 February 2022.

**Key word search**	**Total citations**	**Pubmed search results on 15 Feb 22**
*Exact phrase: “health care” and health outcomes (2000–2022)*	237,912	https://pubmed.ncbi.nlm.nih.gov/?term=health+care+and+health+outcomes&filter=dates.2000-2022
*Exact phrase: “veterinary medicine” and health outcomes (2000–2022)*	3,405	https://pubmed.ncbi.nlm.nih.gov/?term=veterinary+medicine+and+health+outcomes&filter=dates.2000-2022
*Exact phrase: “zoological medicine” and health outcomes (2000–2022)*	2	https://pubmed.ncbi.nlm.nih.gov/?term=zoological+medicine+and+health+outcomes&filter=dates.2000-2022

Utilizing an outcome approach to evaluating veterinary patient care protocols would transform the zoological medicine community by informing efficacy of care protocols, improving quality of care, realizing efficiencies in care, and energizing health care teams.

## Outcomes and Outcome Measures

Outcomes are the results of care or interventions over time and are centered around the patient. Outcomes are measured for each medical condition over the full cycle of care which includes acute care, related complications, rehabilitation, and recurrences (1). Outcomes to measure should involve the health conditions most relevant to patients, or in zoological medicine, most relevant to a particular species, address short-term and longer-term patient health, and cover the full range of interventions that together determine the health and wellness of patients or populations. The overall results matter, not only the outcome of an individual intervention (1, 2). Outcomes can also be decreasing disease prevalence in a given population due to the implementation of preventive measures (2, 3).

For any medical condition or a patient population, success is defined by multiple outcomes. This set of outcomes becomes very broad in scope and includes immediate procedural outcomes, patient return to longer-term functional status, and recovery time. This concept is very familiar to zoo veterinarians who routinely consider the longer-term impacts of care on quality of life. Having multiple outcomes results in outcomes that may compete with each other and these are often weighed against one another. An example of this is weighing a medical intervention that is very safe but less likely to restore full function against one that is less safe but more likely to result in full functionality. Arranging these patient-centered outcomes into three tiers is suggested in the human and companion animal literature. Each tier is further subcategorized by the impact on quality of life (Table 2) (1, 2). Veterinarians engage in patient-centered care through multidisciplinary teams and meaningful discussions with others who share in the care of a patient. This includes a discussion of the evidence, risks, benefits, options, and resource expenditure and ultimately becomes a discussion on quality of life. These discussions can also occur at the population level.

**Table 2:** Outcomes measures hierarchy using a case of dystocia in an antelope that requires surgical intervention.

**Outcome measure**	**Qualifier**	**Outcome example**
1. Health status achieved or retained	Survival	Mortality rate associated with Caesarian section
	Degree of recovery	Reproductive tract intact post-surgery
2. Process of Recovery	Time to recovery and return to normal activity	Assimilation back into herd
	Disutility of care or the treatment process (e.g., diagnostic errors, ineffective care, complications, adverse events, pain/discomfort during treatment, duration of hospital stay)	Surgical site dehiscence; post-operative infection; horn fracture or secondary injury as a result of hospitalization/confinement; prolonged hospitalization,
3. Sustainability of health (patient or population)	Sustainability of health or recovery and nature of recurrences	Aggression from herd when re-introduced; repeat dystocia with subsequent pregnancy
	Long-term consequences of treatment	Ability to deliver live calf and raise it without intervention; genetic diversity maintained in a given population

## Indicators

Health outcomes at the population level may not be observable in the short term. Indicators are used to show progress toward outcomes. Indicators are particularly useful for long-term outcomes and can be evaluative or predictive (4). Lagging indicators, as the name suggests, reflect what has already happened and measure the occurrence and frequency of events that occurred in the past. Examples include morbidity and mortality metrics for a given disease or body weight recorded at monthly intervals. Lagging indicators are output oriented and do not address how well you are doing at preventing disease or a condition such as obesity. Leading indicators, on the other hand, are the activities carried out to prevent a disease or condition. They tell you whether you are likely to achieve an outcome and are typically input oriented. Examples include the utilization of personal protective equipment (PPE), training on the appropriate use of PPE, preventive measures such as vaccination, and measures taken to encourage regular physical activity. Lagging indicators are easier to measure while leading indicators are easier to influence. Lagging indicators do not typically promote behaviors and actions that improve health (5).

Leading indicators are used in the business and economic sectors (6), have more recently been applied in the occupational health and safety sector (5, 7), and are being proposed in the biodiversity conservation sector (4, 6). Using leading indicators in the near-term can help assess likelihood of achieving long-term outcomes. Leading indicators are often predictors of lagging indicators. Lagging indicators reflect the scale of the effort rather than the magnitude of the impact (6).

A balanced approach is recommended which combines lagging and leading indicators, as it is unlikely one overriding indicator can predict or influence a health care outcome. Indicators that are interrelated may, taken together, better monitor and affect health and well-being (5).

## Theory of Change

Leading and lagging indicators are used with recognized frameworks such as a theory of change. Theory of change links actions to outcomes and can be used to enact broad scale health impacts. Drivers are factors that influence an outcome and can be used to identify leading indicators. Broad objectives are articulated into more context-based short-term goals (6).

Conservation Standards, for example, is a framework that uses situation models to document the current state and to document anticipated chains or results and the underlying assumptions. First identify the outcome you want to achieve and then map backwards to best understand the interventions, indicators, outcomes and assumptions that are needed to be successful. Indicators are used to connect short and intermediate outputs with longer term outcomes (8).

**Figure 1:**
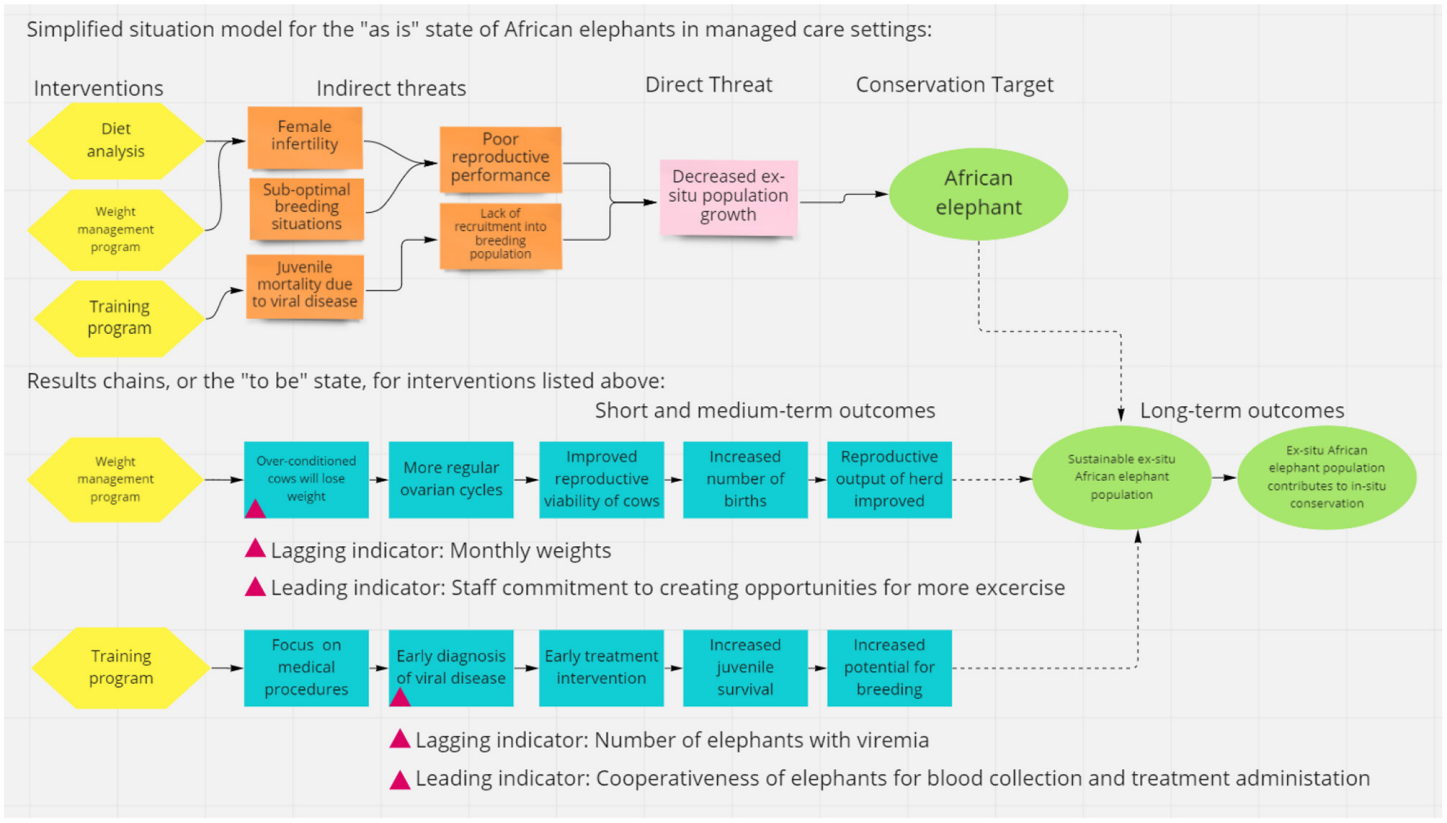
Example of a theory of change model including a situation model, results chains, and lagging and leading indicators created using https://miro.com/.

Theory of change models tend to be visual and promote an iterative process through learning (Figure 1). This helps create a vision and a path that is accessible to all stakeholders and identifies the risks and opportunities that exist when pursuing a broad outcome such as improved health for a population. Theory of change assists in decision making and should be part of an adaptive management approach.

## Conclusion

Generating outcome standards requires patient data. The use of electronic medical records and specifically the increasing use of Species360 ZIMS (Zoological Information Management Software) is generating raw patient data at unprecedented rates (9). The establishment of outcome standards is needed to speed up measurement, allow institutions to collect and share data on outcomes more efficiently, and allow comparisons that will accelerate care improvement (10). The International Consortium for Health Outcomes Measurement (ICHOM) has outlined minimum standard outcome sets and risk factors for human health conditions using a structured process that may serve as a point of reference for the zoological medicine community (10, 11).

These outcome data can become even more impactful when applied to a theory of change framework. Applying outcomes can connect the work of caring for individual animals to managing sustainable populations. This embodies the One Plan approach to species conservation which is the development of management strategies and conservation actions by all responsible parties for all populations of a species, whether inside or outside their natural range (12).

Measuring, reporting, and comparing health outcomes is a strategic approach that can drive better health outcomes for patients and populations. This approach can also drive improvement in care delivered by health teams. Improved outcomes and improved quality of care can be very motivating for teams. This also connects the interventions that improve health to a broader framework that contributes to wildlife conservation.

## Author Contributions

The author confirms being the sole contributor of this work and has approved it for publication.

## Conflict of Interest

The author declares that the research was conducted in the absence of any commercial or financial relationships that could be construed as a potential conflict of interest.

## Publisher's Note

All claims expressed in this article are solely those of the authors and do not necessarily represent those of their affiliated organizations, or those of the publisher, the editors and the reviewers. Any product that may be evaluated in this article, or claim that may be made by its manufacturer, is not guaranteed or endorsed by the publisher.
